# Favorable Outcomes of Chinese HCV-Related Cirrhotic Patients with Sustained Virological Response after Pegylated Interferon Plus Ribavirin Treatment

**DOI:** 10.1155/2017/8061091

**Published:** 2017-01-23

**Authors:** Geng-lin Zhang, You-ming Chen, Ting Zhang, Qing-xian Cai, Xiao-hong Zhang, Zhi-xing Zhao, Chao-shuang Lin, Zhi-liang Gao

**Affiliations:** ^1^Department of Infectious Diseases, The Third Affiliated Hospital, Sun Yat-Sen University, Guangzhou, China; ^2^Guangdong Key Laboratory of Liver Disease Research, The Third Affiliated Hospital, Sun Yat-Sen University, Guangzhou, China; ^3^Department of Ultrasound, The Third Affiliated Hospital, Sun Yat-Sen University, Guangzhou, China; ^4^Key Laboratory of Tropical Disease Control, Sun Yat-Sen University, Ministry of Education, Guangzhou, China

## Abstract

Few studies have conducted follow-up investigations of the clinical course in HCV-related cirrhotic patients who achieved a sustained virological response (SVR) with pegylated interferon plus ribavirin treatment (PegIFN + RBV). We investigated the clinical course and laboratory data in a prospective cohort study enrolling HCV-related cirrhotic patients who received PegIFN + RBV between August 2008 and July 2013 in China. Complete blood counts, liver function tests, and HCV-RNA were serially examined. Liver-related complications were recorded. To detect hepatocellular carcinoma (HCC), alpha-fetoprotein assays, and ultrasound scans were repeated at 6-month intervals. Twenty-five patients were enrolled, including 8 patients with decompensation events before treatment. Eighteen patients achieved SVR with a mean follow-up period of 25.78 months. During the follow-up period, only one patient exhibited HCV-RNA positivity and no decompensation events were detected, but 4 patients developed HCC after SVR. APRI decreased more in patients with SVR than in patients with non-SVR (median, −1.33 versus 0.86, *P* < 0.001). The albumin levels and platelet counts significantly increased during the follow-up period after SVR (44.27 ± 4.09 versus 42.63 ± 4.37, *P* = 0.037 and 173.89 ± 87.36 versus 160.11 ± 77.97, *P* = 0.047). These data indicated that HCV-related cirrhotic patients with SVR after PegIFN + RBV may have a favorable clinical course and improvements in laboratory data. Moreover, HCC should be monitored.

## 1. Introduction

Approximately 25–50 million Chinese were infected with hepatitis C virus (HCV) [1]. Without treatment, 16% of patients with HCV progress to liver cirrhosis within 20 years after infection, and 41% develop liver cirrhosis within 30 years [2]. As the patients infected with HCV age, the risk of developing life-threatening complications (decompensated cirrhosis or hepatocellular carcinoma) is expected to increase [3]. The annual risk of developing decompensated liver diseases has been shown to be 4% in cirrhotic patients. The annual mortality is 13% for patients with decompensated liver disease, and the ten-year survival rate is only 25% [4]. Thus, these cirrhotic patients infected with HCV make a significant burden on public health.

Before the introduction of direct-acting antiviral agents (DAAs), the combination of pegylated interferon and ribavirin (subsequently referred to as PegIFN + RBV) was the approved treatment for chronic hepatitis C (CHC) [5]. The incidence of developing hepatic events (decompensation, hepatocellular carcinoma (HCC) and death) reduced in patients with sustained virological response (SVR) [6–8]. A report showed that interferon therapy could be associated with a reduction of HCC development even in patients without SVR [8]. Moreover, successful antiviral therapy in selected patients waiting for liver transplantation could delay the disease progression and can prevent the transplanted liver HCV reinfection, subsequently leading to a decrease of posttransplant morbidity and mortality [9–12]. Therefore, eradication of HCV in this population should be urgently considered.

To date, data focused on this topic in Chinese patients are scarce. Thus, to determine the impact of SVR on the clinical outcomes of cirrhotic patients, we conducted this retrospective analysis of data from a prospective cohort study enrolling Chinese patients with HCV-related cirrhosis, treated with PegIFN + RBV and followed up according to standardized criteria. We serially assessed changes in the laboratory data from patients achieving SVR.

## 2. Patients and Methods

### 2.1. Study Design

Chinese patients with HCV-related liver cirrhosis who were previously untreated were enrolled into our study to receive PegIFN + RBV treatment at our department between August 2008 and July 2013. Criteria for admission to our study included a positive serum test of anti-HCV and HCV-RNA, cirrhosis proved by liver biopsy, or evidence of an irregular and nodular liver by ultrasonography or magnetic resonance imaging (MRI) together with impaired liver synthetic function. Cirrhotic patients with a history of decompensation events (including ascites, sepsis, variceal bleeding, and hepatic encephalopathy) were included if the Child-Turcotte-Pugh (CTP) score < 9 at enrollment. Patients who met with any of the following criteria were excluded: (1) coexisting with other liver disorders, (2) a positive test of anti-HIV, (3) active drug users or ongoing alcohol consumption, (4) patients with an uncontrolled psychiatric disease, (5) pregnancy, (6) history of organ transplantation, (7) CTP score ≥ 9, and (8) laboratory values for creatinine ≥ 1.5 mg/dL, absolute neutrophil counts < 1000/mL, platelet counts < 50,000/mL, or hemoglobin < 10.0 g/dL.

Patients received a combination of PegIFN-a-2a (Pegasys, Roche, Basel, Switzerland) plus daily RBV for a duration of 48 weeks. Patients were initially treated with PegIFNa-2a (180 *μ*g/week) plus RBV (900 mg/day), and then the dose was decreased or adjusted as a function of hematologic tolerance. Growth factor use (erythropoietin, granulocyte colony-stimulating factor, and/or recombinant human interleukin-11) was used to maintain adherence to therapy. Treatment was discontinued if HCV-RNA loads at week 12 dropped less than 2 log compared with baseline values, or if HCV-RNA loads still can be detected at week 24, or viral breakthrough existed. All patients were required to undergo the follow-up program after termination of treatment. The study was approved by the Ethics Committee of our hospital. All patients provided written informed consent. The study conformed to the ethical guidelines of the 1975 Declaration of Helsinki.

### 2.2. Assessment of Response to Therapy

A SVR was defined as HCV-RNA negativity determining by the Roche Amplicor™ HCV test (the lower limit of detection was 15 IU/mL) for more than 6 months after therapy, and any other outcome was considered as nonsustained virological responses (non-SVR). Negativity of serum HCV-RNA was assessed at treatment week 4 (rapid virological response, RVR), at treatment week 12 (early virological response, EVR), at treatment week 24 (delayed virological response, DVR), and at the end of treatment (EOT). After EOT, patients who tested positive for HCV-RNA during the follow-up were defined as relapsers.

### 2.3. Laboratory and Imaging Assessment

Evaluation of the patients involved a medical history, a physical examination, and laboratory tests (including the complete blood counts, biochemical tests). Anti-HCV was assessed using the ARCHITECT system (Abbott Diagnostics, Abbott Park, IL, USA). HCV-RNA loads were measured using the Roche Amplicor HCV test (the lower limit of detection was 15 IU/mL.) according to the manufacturer's instructions. HCV-RNA was detected at baseline (BL), week 4, week 12, week 24, week 36, EOT, and 24 weeks after treatment and the follow-up period. INNO-LiPA HCV II kit assay was used to determine the HCV genotype. Liver biopsy specimens were evaluated by two liver pathology specialists who were blinded to the etiology.

### 2.4. Follow-Up

The length of the follow-up period was calculated from the starting date of certification of SVR to the last follow-up visit. Complete blood counts, liver function tests, HCV-RNA, and physical examinations were performed every 6 months during follow-up and at the last visit. Liver-related events (ascites, upper gastrointestinal bleeding, and hepatic encephalopathy) were recorded. Ascites was diagnosed by physical examination and/or ultrasound detection. Portosystemic encephalopathy was defined by clinical manifestations. Endoscopy was used to confirm the source of gastroesophageal bleeding if needed. To detect HCC, alpha-fetoprotein (AFP) assays and ultrasound scans were repeated every 6 months. If HCC development was suspected, MRI was performed. HCC was diagnosed according to the guidelines of the European Association for the Study of the Liver [13].

### 2.5. Statistical Analysis

Continuous variables are expressed as mean and standard deviation or median and range, and categorical variables are reported as the absolute and relative frequencies. The Wilcoxon signed-rank test and analysis of variance (ANOVA) were used to analyze the data. Comparisons between groups were conducted by the Mann–Whitney *U* test or Student's *t* test for continuous variables and Fisher's exact probability test for categorical data. Ratios were examined by Pearson's chi-square test. A *P* value less than 0.05 was considered statistically significant. All statistical analyses were performed using the SPSS 13 software package (SPSS Inc., IL, USA).

## 3. Results

### 3.1. Patient Characteristics

Of a total of 28 consecutive HCV-related cirrhotic patients who met the diagnostic criteria, 3 were excluded from the final analysis (1 withdrew for intolerance after the first injection, and 2 were lost to follow-up). Finally, 25 patients completed the therapy and were assigned to the study group. Four patients were diagnosed with cirrhosis by liver biopsy, whereas the other 21 patients were diagnosed clinically before enrollment. The mean age was 48.76 ± 9.53 (range: 33–65) years; there were 15 (15/25, 60%) males. The majority of patients (15/25, 60%) were infected with HCV genotype 1. Among them, 8 (32%) patients had a history of decompensation events (6 patients with ascites, 2 patients with variceal bleeding). At baseline, 2 patients had Child B class cirrhosis, and 23 patients had Child A class cirrhosis. Eighteen patients achieved SVR after a course of therapy, resulting in a SVR rate of 72% (18/25). Patients with different treatment outcomes (18 patients with SVR, 7 patients with non-SVR) were similar in terms of the demographic, biochemical and virological data at baseline (Table 1). Nine patients in the SVR group received spleen interventions before therapy (6 with splenectomy and 3 with splenic embolization); however, no participants in the non-SVR group received such therapy, resulting in a difference in platelet counts. The baseline characteristics of the patients based on the response to treatment are shown in Table 1.

### 3.2. Response to Therapy

Fatigue, a prolonged flu-like syndrome, and abnormalities of hematocytes were the common subjective adverse events. Symptomatic treatment and growth factor use were encouraged to maintain adherence to therapy. 25 patients tolerated the treatment well and completed it. Eighteen patients achieved SVR (18/25, 72%). In the subgroup of patients with decompensation events, 6 achieved SVR (6/8, 75%). The rate of SVR according to the HCV genotype was 60% (9/15) for genotype 1 patients and 90% (9/10) for non-genotype 1 patients. Nonresponse (NR) was observed in 4 (16%) patients. At treatment week 4, 13 patients (52%) had undetectable HCV-RNA. At treatment week 12, 20 patients (80%) had undetectable HCV-RNA. At treatment week 24, 21 patients (84%) had undetectable HCV-RNA. During the remaining treatment, 3 (12%) patients developed virologic breakthrough and HCV-RNA became positive at treatment weeks 36, weeks 36, and weeks 48, respectively. Eighteen patients (72%) achieved an EOT response and remained HCV-RNA negative through week 24 after termination of therapy. Therefore, SVR was observed in 18 (72%) patients.

### 3.3. Long-Term Outcomes

The eradication of HCV infection was indicated with a significantly lower rate of cirrhosis-related complications, HCC, and deaths. The follow-up period initiated from the time patients achieved SVR. The average duration of follow-up period was 25.78 ± 13.20 months. HCV-RNA was examined every 6 months using the Roche Amplicor HCV test during the follow-up period. Only one patient (5%) developed HCV-RNA positivity at 6 months after the date of SVR confirmation. The long-term clinical outcomes were assessed at every visit. No decompensation events were detected in the patients with SVR. Moreover, no patients died. However, 4 patients (2 males and 2 females) with SVR developed HCC during the follow-up period. Three of these patients had a genotype 1 HCV infection, and the other patient had a genotype 3 infection. Two of these patients had episodes of decompensation events before enrollment. Their baseline AFP levels were 9.95 ± 1.54 ng/mL, which is in the normal range (AFP reference range < 20 ng/mL). Moreover, the AFP levels at the time of the HCC diagnosis were also normal (6.63 ± 1.98 ng/mL). The confirmation time of HCC diagnosis was different; two patients were diagnosed at the date of confirmation of SVR, one was diagnosed within the first year, and the other was diagnosed in the third year after confirmation of SVR. All patients had a single lesion with a mean diameter less than 3 cm that was first detected by ultrasound and then confirmed by MRI. Two cases underwent surgical resection, and others received radiofrequency ablation.

#### 3.3.1. ALT Values, Absolute Platelet Counts, Albumin Values, and AST-to-Platelet Ratio Index (APRI) Levels at the Initiation of Therapy and at the End of Follow-Up

**Figure 1:**
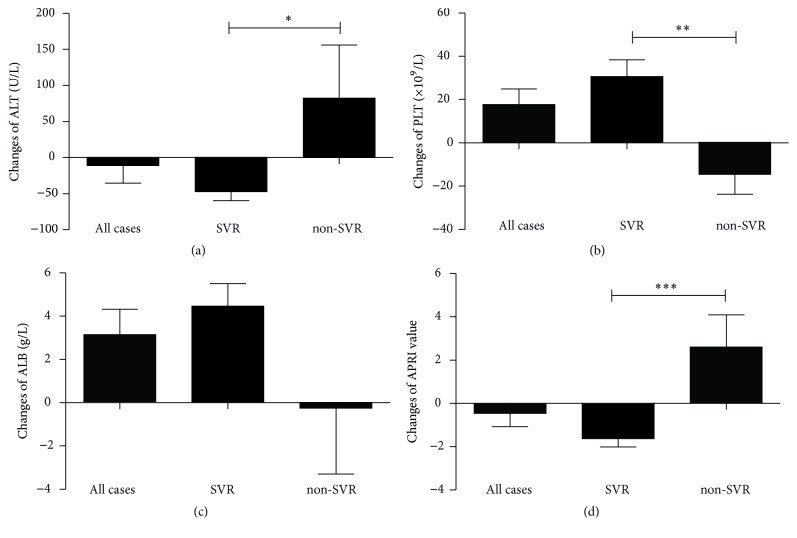
Changes in ALT values, absolute platelet counts, albumin values, and APRI levels in each group based on different treatment outcomes. The ALT levels (a) and APRI values (d) significantly reduced in the SVR patients compared with the non-SVR patients. Platelet counts (b) and albumin levels (c) increased in the patients with SVR and decreased in the non-SVR patients. *P* values represent comparisons between values at the initiation of treatment and at the last visit using the Wilcoxon signed-rank test or Student's *t*-test. ALT: alanine aminotransferase; PLT: platelet counts; ALB: albumin; APRI: aspartate aminotransferase to platelet ratio index; SVR: sustained virological response. ^*∗*^*P* < 0.05; ^*∗∗*^*P* < 0.01; ^*∗∗∗*^*P* < 0.001.

Changes in ALT values between the end of follow-up and the initiation of therapy significantly decreased in the SVR group than patients with non-SVR (*P* = 0.025) (Figure 1(a)). The decreased value in each group was −19 U/L (−141, 512) in all patients, −26.5 U/L (−141, 7) in patients with SVR, and 0 U/L (−34, 512) in patients with non-SVR. The platelet counts increased in patients with SVR and decreased in non-SVR patients (*P* = 0.003) (Figure 1(b)). The extent of change in each group was 18 ± 36.48 in all patients, 30.67 ± 32.60 in patients with SVR, and −14.57 ± 24.36 in patients with non-SVR. A significant difference was observed in the values between SVR and non-SVR patients (*P* = 0.003), indicating that the platelet counts elevated only in patients who gained the eradication of HCV. The albumin levels elevated in SVR patients and decreased in non-SVR patients (*P* = 0.215) (Figure 1(c)). The extent of change in albumin values in each group was 3.2 g/L (−17.5, 14.2) in all patients, 3.35 g/L (−1.1, 14.2) in patients with SVR, and 1.8 g/L (−17.5, 6.5) in patients with non-SVR. The AST-to-platelet ratio index (APRI) was used to evaluate liver fibrosis. High APRI values can represent the progression of liver fibrosis. APRI was calculated according to the published formula [14, 15]. In our study, the APRI values declined significantly in SVR patients compared with non-SVR patients (*P* < 0.001) (Figure 1(d)). The extent of change in APRI levels in each group was −1.09 (−6.90, 10.4) in all patients, −1.33 (−6.90, 0.04) in patients with SVR, and 0.86 (−1.09, 10.4) in patients with non-SVR. The APRI value increased in almost all patients with non-SVR (6/7, 86%); however, the APRI value only increased in one patient with SVR (5% versus 86%, *P* < 0.001), suggesting that cirrhosis may be resolved in patients with HCV eradication.

#### 3.3.2. Serial Changes in ALT Values, Absolute Platelet Counts, Albumin Values, and APRI Levels in Patients with SVR

**Figure 2:**
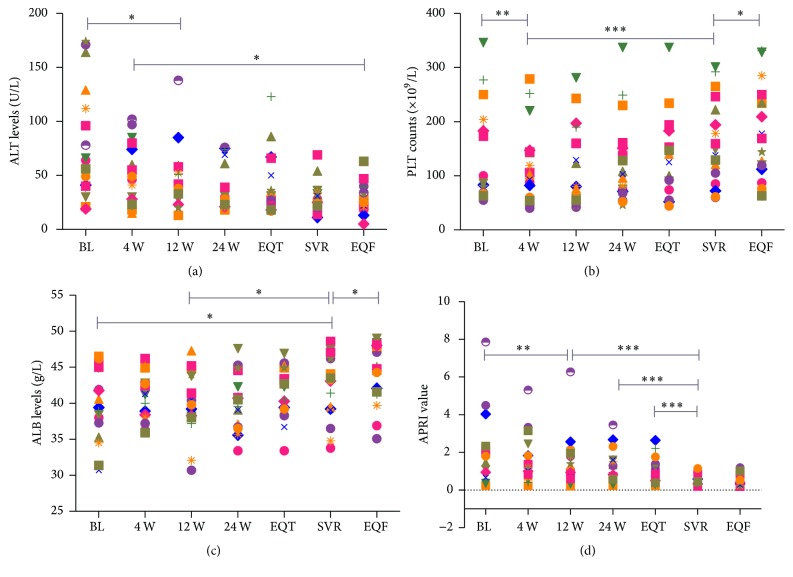
Serial changes in ALT values, absolute platelet counts, albumin values, and APRI levels in patients with SVR. ALT levels decreased progressively after the initiation of treatment until the end of the follow-up period (a). Platelet counts markedly decreased at treatment week 4 and then gradually increased (b). Albumin levels markedly increased during the follow-up period after the certification of SVR (c). APRI values decreased progressively during the therapy and tended to decline continuously throughout the follow-up (d). ALT: alanine aminotransferase; PLT: platelet; ALB: albumin; APRI: aspartate aminotransferase to platelet ratio index; BL: baseline; 4 W: treatment week 4; 12 W: treatment week 12; 24 W: treatment week 24; EOT: end of treatment response; SVR: sustained virological response; EOF: end of follow-up. ^*∗*^*P* < 0.05; ^*∗∗*^*P* < 0.01; ^*∗∗∗*^*P* < 0.001.

We analyzed the serial changes in ALT values, absolute platelet counts, albumin values, and APRI levels in the 18 patients who achieved SVR. The ALT values decreased progressively after the initiation of treatment until the end of the follow-up period. The ALT values declined rapidly 12 weeks after the beginning of treatment and gradually until confirmation of SVR by 36.5 U/L (13, 138) (*P* = 0.033) and 27.5 U/L (11, 69) (*P* = 0.001), respectively. Then, the ALT levels nearly returned to normal and continued to decrease gradually until the end of the follow-up period (29.56 ± 13.5 U/L) (Figure 2(a)). The mean platelet count was 136.5 × 10^9^/L (55, 346) at baseline. The platelet counts markedly decreased 4 weeks after the initiation of treatment by 31 ± 39.72 (×10^9^/L) (*P* = 0.002) and gradually increased thereafter with a SVR of 160.11 ± 77.97 (*P* < 0.001). Moreover, the platelet counts continued to increase gradually until the end of follow-up compared with the value at SVR (173.89 ± 87.36, *P* < 0.047) (Figure 2(b)). The albumin levels did not change significantly during the first 12 weeks after the initiation of treatment (*P* = 0.922), but they increased gradually thereafter with a significant difference at SVR compared with week 12 (42.63 ± 4.37 versus 40 ± 4.26 g/L, *P* = 0.014). More interestingly, albumin levels markedly increased during the follow-up period after certification of SVR (44.27 ± 4.09 versus 42.63 ± 4.37 g/L, *P* = 0.037). The mean albumin level was 40.12 ± 5.02 at baseline, 44.00 ± 4.26 at week 12, 42.63 ± 4.37 at SVR, and 44.27 ± 4.09 at the last follow-up, respectively (Figure 2(c)). The APRI value decreased progressively after initiation of treatment until the end of follow-up. The APRI value was 1.91 (0.24–7.86) at baseline and 1.35 (0.22–6.27) at week 12 with a significant difference compared with baseline (*P* = 0.006) and then gradually decreased to a lowest value at SVR 0.58 (0.19–1.15) compared to the baseline value (*P* < 0.001). The APRI levels tended to decline continuously throughout the follow-up period to a value of 0.37 (0.18–1.19), but the difference was not significant (*P* = 0.196) (Figure 2(d)).

## 4. Discussion

Directly acting antiviral agents (DAAs) may be the first choice for HCV-related cirrhotic patients in many western countries. Studies have shown that DAAs can overcome the drawbacks of interferon based therapy in HCV-related cirrhotic patients. Data on compensated cirrhotic patients showed SVR rates more than 90%, and a little lower SVR rate in decompensated patients with an optimal safety profile [16]. Moreover, a recent study reported that the rate of liver transplant wait-listing for patients with decompensated cirrhosis decreased by over 30% using DAAs therapy [17]. However, in China, clinical trials focusing on DAA are incomplete, and these agents are not approved for clinical use. Because of the incidence of liver decompensation, which is approximately 2–6% per year [18], cirrhotic patients should be treated in an urgent manner. Therefore, the combination of PegIFN + RBV remains the standard of care for cirrhotic patients. Patients with advanced liver disease typically have a poor response to PegIFN + RBV. Pooled metaestimates for SVR rates in cirrhotic patients revealed 17% with PegIFN + RBV [19]. Our results showed a SVR rate of 72%, slightly high for cirrhotic patients as 32% patients in our study had a history of decompensation events. This disparity may be interpreted by the different host genetic factors which could affect the treatment response. The single nucleotide polymorphisms near the interleukin (IL) 28B gene have been recently indicated to be linked with treatment outcomes and may be a good indicator of SVR. A cross-sectional observational study conducted in China showed that the majority of patients (84.1%) carried IL-28B genotype CC (rs12979860) [20]. The better efficacy of antiviral therapy in our study may be attributed to the favorable genotype of IL-28B polymorphisms.

HCV genotype definitely plays an important role in the response to PegIFN + RBV. The SVR rate was approximately 40% in North America and 50% in Western Europe patients infected with HCV genotype 1. Higher SVR rates were obtained in patients infected with other genotypes (up to approximately 80%) [21]. But, these data were achieved from patients with chronic hepatitis C. Consistent with previous reports [22, 23], HCV genotype 1 patients in our study developed lower SVR rates compared with other genotypes (60% versus 90%). Previous studies have reported that the SVR rate can be predicted by measuring of HCV-RNA at treatment week 12. Our results were similar to those of previous studies. In our study, 90% of patients (18/20) who achieved EVR finally had SVR after treatment, suggesting a strong positive predictive value for SVR. Several reports evaluating long-term follow-up studies of chronic hepatitis C patients achieving SVR have indicated that the incidence of relapse of HCV-RNA is relatively low, although relapse is not rare [24, 25]. During the mean follow-up period of 25.78 months, the reappearance of HCV-RNA was detected in only one patient, suggesting the persistent presence of undetectable HCV-RNA, which might result in decreased hepatic inflammation.

The mortality of cirrhotic patients is determined majorly by decompensation complications or HCC. Therefore, eradication of HCV by successful antiviral therapy may halt disease progression and HCC development, finally lowering disease-related mortality. A recent Japanese study demonstrated that SVR with successful antiviral therapy decreased the risk ratio for the overall mortality and disease-related mortality of HCV patients [26]. In addition, achieving SVR decreased the risk of HCC, liver decomposition, and all-cause mortality in patients with cirrhosis [27, 28]. Achieving SVR before liver transplantation in patients with advanced cirrhosis has been shown to reduce the risk for posttransplant HCV virus recurrence, which is known to limit graft and overall survival [29]. However, there are little data concerning the long-term outcomes in patients with HCV-related liver cirrhosis who achieved SVR, especially in Chinese patients. In our study, we demonstrated a favorable clinical course in southern Chinese patients who achieved SVR. In the follow-up period, no decompensation events were recorded. Furthermore, no deaths occurred. Therefore, because of its potential benefits, PegIFN + RBV should be an alternative choice and be encouraged in selected cirrhotic patients.

The risk of HCC was considered to decrease among patients who achieved SVR after PegIFN + RBV therapy [27, 30]. However, several recent reports revealed that viral eradication induced by DAAs maybe did not reduce the risk of HCC development in cirrhotic patients; furthermore patients who were previously treated for HCC still had a high risk of recurrence [31, 32]. In our study, 4 patients (22%) who achieved SVR developed HCC within the first 3 years during the follow-up. A previous study reported that the AFP levels after interferon treatment were correlated with the occurrence of HCC among patients with SVR [33]. AFP levels greater than 6 ng/mL have been shown to be associated with an increasing risk of HCC. Similarly, this study showed that the AFP levels were more than 6 ng/mL at baseline, even at the time of SVR confirmation. Although the AFP level was normal, 4 patients were suspected of HCC development by ultrasound, and confirmed by MRI. Because our study was a retrospective study enrolling small cohort of patients, causal relationships could not be established. Prospective studies are needed to define the relationship between HCC and SVR and to determine the best surveillance options for cirrhotic patients.

There is little information available concerning changes in the laboratory parameters of patients with HCV-related cirrhosis who gained SVR. In our study, ALT levels and APRI levels reduced in SVR patients in the follow-up period. Absolute platelet counts and albumin values declined in patients with non-SVR and increased in patients who achieved SVR. These data indicated that the progression of liver fibrosis differs between SVR patients and non-SVR patients. Several studies showed that histological improvement was achieved in chronic hepatitis C patients who achieved SVR after antiviral therapy [34, 35]. In a subgroup from the HALT-C trial, patients with advanced fibrosis or cirrhosis treated with low-dose PegIFN showed both a histological improvement and a reduction in portal pressure [36]. The APRI was considered to be a surrogate marker of fibrosis. The change in APRI in our study appeared to support this hypothesis. Our results were based on laboratory data but not on liver biopsy, providing indirect data regarding the improvement of hepatic fibrosis.

Furthermore, serial changes in ALT values, absolute platelet counts, albumin values, and APRI levels in patients with SVR showed that the ALT levels decreased for only 12 weeks after the initiation of treatment and returned to nearly normal during the study period. The absolute platelet counts continued to elevate significantly. Thus, serial changes in the absolute platelet counts may be used to estimate the long-term improvement of liver fibrosis after SVR. Consistent with the absolute platelet counts, the albumin values elevated significantly during the follow-up period. Previous long-term follow-up studies revealed no differences in ALT values, albumin values, or absolute platelet counts at the last visit point compared with 24 weeks after the termination of IFN treatment [37, 38]. However, our results demonstrated gradual increases in platelet counts after treatment. Previous reports have revealed that ALT levels normalized in the majority of CHC patients after SVR [38], although only a few studies have reported this finding in cirrhotic patients.

To our knowledge, rare studies have conducted a follow-up of longitudinal changes in APRI levels in HCV-infected cirrhotic patients who achieved SVR in a long-term study. Liver biopsy is the most reliable approach to evaluate the fibrotic stage, although this procedure has some limitations, such as sampling variability, procedural discomfort, and added cost. A previous study showed that APRI could be used to distinguish between F0-F1 and F2–F4 fibrosis [14]. Moreover, APRI was recently recommended by the WHO to assess the stage of fibrosis for making decisions regarding antiviral therapy [15]. The APRI levels were reduced significantly at treatment week 12 and then gradually decreased throughout the entire study period, suggesting a histological improvement in patients with SVR. As we know, rare reports have demonstrated an improvement of liver fibrosis using liver biopsy in cirrhotic patients with SVR during the long-term follow-up period, which may be due to the increasing risk of bleeding. We demonstrated this improvement by monitoring the changes of platelet counts and APRI, the consequential index of fibrosis.

Our study has some limitations. Firstly, histological confirmation by liver biopsy was only available in a few patients before treatment; the diagnosis of cirrhosis and its progression were determined by clinical evidence in most patients. Secondly, this was a descriptive study and the follow-up period (median of 25.6 months) was not long enough. In addition, among 18 patients with SVR, 4 patients developed HCC during the follow-up period, resulting in a relatively high incidence of HCC development. Therefore, large sample cohort studies are needed for further confirmation.

We reported a favorable clinical outcome in patients who gained SVR after treatment with PegIFN + RBV. HCV-RNA remained negative in most patients, and the laboratory parameters including APRI improved during the follow-up period. Thus, anti-HCV treatment should be urgently considered when the diagnosis of cirrhosis was established. Ultimately, HCC development could be detected in several SVR patients. Therefore, HCV clearance does not mean cure, and HCC should be closely monitored.

## Figures and Tables

**Table 1:** Baseline characteristics of 25 patients with HCV-related cirrhosis stratified based on different responses.

Group	All patients (*n* = 25)	SVR (*n* = 18)	Non-SVR (*n* = 7)	*P* (SVR versus Non-SVR)
Age (years)	48.76 ± 9.53	46.89 ± 9.14	53.57 ± 9.43	0.117
Sex (male, *n*)	15	11	4	0.856
BMI	23.05 ± 2.96	23.12 ± 3.26	22.86 ± 2.21	0.852
Compensated (*n*)	17	12	5	0.819
CTP score (5/6/7)	19/4/2	14/3/1	5/1/1	0.769
HGB (g/L)	133.08 ± 17.11	134.11 ± 16.49	130.43 ± 19.71	0.639
Neutr (×10^9^/L)	2.30 ± 0.93	2.42 ± 1.05	1.98 ± 0.43	0.154
PLT (×10^9^/L)	86 (55–346)	136.5 (55–346)	77.28 ± 14.53	0.085
ALT (U/L)	55 (19–174)	60 (19–174)	50 (35–68)	0.449
AST (U/L)	72 (24–217)	70.5 (24–217)	73 (37–157)	0.966
Albumin (g/L)	39.66 ± 5.40	40.12 ± 5.02	38.50 ± 6.56	0.513
Tbil (*µ*mol/L)	18.55 ± 8.67	18.35 ± 9.40	19.09 ± 7.05	0.853
APRI	1.98 (0.24–7.86)	1.90 (0.24–7.86)	2.24 (1.05–5.45)	0.270
HCV-RNA (IU/mL)	6.22 ± 1.00	6.05 ± 1.14	6.65 ± 0.23	0.183
HCV-RNA > 800,000 (IU/mL)	19	12	7	0.137
Genotype (1/2/3/6, *n*)	15/3/3/4	9/3/2/4	6/0/1/0	0.323

SVR: sustained virological response; BMI: body mass index; CTP: Child-Turcotte-Pugh score; Neutr: neutrophil; ALT: alanine aminotransferase; AST: aspartate aminotransferase; Tbil: bilirubin; APRI: aspartate aminotransferase to platelet ratio index. Data are shown as the mean ± SD or median (range).

## Abbreviations

APRI: Aspartate aminotransferase to platelet ratio index
PegIFN: Pegylated interferon
RBV: Ribavirin
SVR: Sustained virological response.
